# Aroma Characteristics of Lavender Extract and Essential Oil from *Lavandula angustifolia* Mill.

**DOI:** 10.3390/molecules25235541

**Published:** 2020-11-26

**Authors:** Xiangyang Guo, Pu Wang

**Affiliations:** 1 College of Chemistry and Environmental Engineering, Shenzhen University, Shenzhen 518060, China; 2 Department of Agronomy, School of Life Sciences, Inner Mongolia University, Hohhot 010020, China

**Keywords:** lavender (*Lavandula angustifolia* Mill.), extracts and essential oils, volatiles, gas chromatography-mass spectrometry (GC-MS), sensory evaluation, odor characteristics

## Abstract

Lavender and its products have excellent flavor properties. However, most studies focus on the aroma profiles of lavender essential oil (LEO). The volatiles in lavender extracts (LEs), either in volatile compositions or their odor characteristics, have rarely been reported. In this study, the odor characteristics of LEs and LEO were comprehensively investigated by gas chromatography-mass spectrometry (GC-MS), coupled with sensory evaluation and principal chemical analysis (PCA). In addition, the extraction conditions of lavender extracts from inflorescences of *Lavandula angustifolia* Mill. were optimized. Under the optimal conditions of extraction, twice with 95% edible ethanol as the solvent, the LEs tended to contain the higher intensity of characteristic floral, herbal and clove-like odors as well as higher scores of overall assessment and higher amounts of linalool, linalool oxides I and II, linalyl acetate, lavandulyl acetate and total volatiles than LEO. PCA analysis showed that there were significant differences on the odor characteristics between LEO and LEs. The LEO, which was produced by steam distillation with a yield of 2.21%, had the lower intensity of floral, clove-like, medicine-like, pine-like and hay notes, a lower score of overall assessment and lower levels of linalool oxides I and II, linalyl acetate, lavandulyl acetate and total volatiles compared with LEs, whereas the relative contents of linalool and camphor in LEO were significantly higher than that in LEs. Furthermore, the earthy, green and watery odors were only found in LEO. Concerning the odor characteristics and volatile compositions, the LEs had better odor properties than LEO. These results provided a theoretical basis for the industrial preparation of lavender-related products.

## 1. Introduction

Lavender (*Lavandula angustifolia* Mill.) is a perennial, medicinal and aromatic plant that is native to the Mediterranean region and has been widely cultivated in China, especially in Yili, Xinjiang [1]. Lavender possesses excellent biological activities such as antibacterial [2], anti-inflammatory [3,4], anticancer [5] and antioxidant effects [6,7], which are beneficial to human health. Lavender is also effective as an adjunctive therapy in alleviating agitated behaviors of patients with dementia [8]. In addition, lavender-related products, such as lavender essential oil and extracts produced therefrom, show outstanding aroma properties with a pleasant floral note and are widely used in perfume, food flavoring and cosmetic industry as well as aromatherapy [7,9,10]. The volatile compounds in a certain composition and proportion are the material basis for the health benefits of lavender, subsequently determine the odor profiles of lavender-related products, and thus influence the sensory characteristics and application performance of lavender [3]. Up to now, more reports focus on the analysis of volatile compositions in lavender essential oil [1] or the effects of variety and growth on essential oil properties [11]. However, few studies have been conducted on the aroma compositions and flavor characteristics of lavender extracts.

The extraction solvents and extraction times are the vital factors affecting the quality of extracts [12,13]. Generally, natural plant extracts are prepared with purified water or edible ethanol as extraction solvents, allowing for good solubility and safety, mainly for application in making beverages and producing food flavoring [12]. Lavender extracts have a relatively real flavor similar to that of raw materials, which lays the basis for their good application performance in the food industry. The odor property of lavender extracts provides supporting data and shows similarities and differences with essential oils. Therefore, it is worth studying the volatile compositions and the related odor characteristics of lavender extracts.

Steam distillation (SD) is commonly used for the extraction of essential oils and enrichment of volatiles, including those in lavender [1,11]. The odorants of essential oils and extracts could be separated and qualitatively analyzed using gas chromatography-mass spectrometry (GC-MS) [14]. Solid-phase microextraction (SPME), which is a relatively simple method and does not produce artifacts, is commonly used to obtain the aroma compounds of complex matrix samples [15,16].

In the present work, the volatile compositions and odor profiles of lavender extracts and essential oil were comparatively investigated by GC-MS coupled with sensory evaluation, and the extraction conditions of extraction solvents and extraction times for lavender extracts were also optimized. The present research would provide scientific foundation for the production and quality control of lavender-related products.

## 2. Results and Discussion

### 2.1. Odor Property of Lavender Extracts

Regarding the importance of extraction solvents and extraction times for the quality of lavender extracts (LEs), the two factors were optimized based on the odor properties of LEs, which were evaluated by sensory evaluation coupled with volatile analysis by GC-MS.

#### 2.1.1. Effect of Extraction Solvent on the Volatile Compositions of LEs

**Figure 1:**
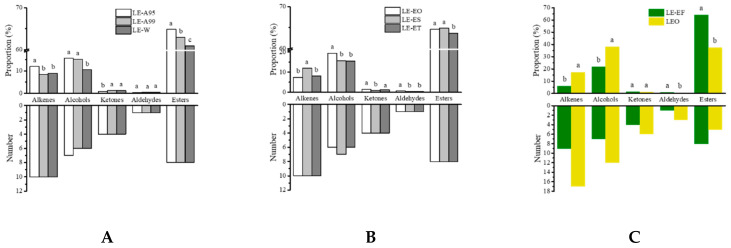
Aroma profiles of lavender extracts and lavender essential oil. (**A**) The proportion and number of volatile categories from lavender extracts with different extraction solvents. (**B**) The proportion and number of volatile categories from lavender extracts with different extraction times. (**C**) The proportion and number of volatile categories from lavender essential oil and lavender extracts under the optimized extraction conditions. LE-W—extraction with purified water; LE-A95—extraction with 95% edible ethanol; LE-A99—extraction with 99% edible ethanol; LE-EO—extraction once; LE-ES—extraction twice; LE-ES—extraction three times; LE-EF—the final lavender extracts; LEO—the lavender essential oils.

A total of thirty-three volatile compounds were identified by GC-MS. In detail, a number of 32, 32 and 33 volatile compounds were found in LE-W (extraction with purified water), LE-A99 (extraction with 99% edible ethanol) and LE-A95 (extraction with 95% edible ethanol), respectively, and the total amounts of volatiles in each sample were 83.60%, 90.42% and 96.40%, respectively. These identified volatiles belonged to various chemical groups, including alkenes, alcohols, ketones, esters and aldehydes. The number and proportion of these chemical categories varied in the three samples (Figure 1A) and the majority were esters, alcohols and alkenes with fruity, floral or spicy notes.

The ester compounds dominated the volatile compositions in the three samples, and eight volatile compounds were identified in each sample, accounting for 60.91%, 62.91% and 64.85% of the total amount of volatiles in LE-W, LE-A99 and LE-A95, respectively. As displayed in Table 1, linalyl acetate and lavandulyl acetate with floral odors reminiscent of lavender [1] were of high abundance and their total amounts were more than 57% of the total amount of volatiles in the three samples, and the highest amount was 58.50% in LE-A95 and the lowest amount was 57.32% in LE-W. The number of alkene compounds was the highest, and ten chemical compounds were identified in each sample, but the relative content was significantly different among the three samples, being the highest at 11.99% in LE-A95 and the lowest at 8.45% in LE-A99. (*E*)-β-Caryophyllene and (*E*)-β-farnesene, which were of spicy and floral odors, respectively [17,18], were the two abundant alkenes with a relative content higher than 1%. The LE-W contained the lowest number and amount of alcohol compounds (6 and 10.49%, respectively). No significant difference was observed on the amount of alcohols between LE-A95 and LE-A99 (15.66% and 15.20%, respectively). 1-Octen-3-ol with earthy odor [19] was only identified in LE-A95. Linalool was the most abundant alcohol in the three samples and was considered as the key aroma-active compound imparting a floral note for lavender [20,21]. Volatiles in LE-A95 possessed the strongest intensity of floral odor, most reasonably due to the highest amount of linalool (12.51%), and were significantly higher than that in other samples. Besides linalool, the highest amounts of linalool oxides (I and II) with floral odors were found in LE-A95. Four ketone compounds were identified in each sample, and the lowest amount of ketones was found in LE-A95, while the highest amount of ketones was found in LE-W (0.83% and 1.29%, respectively). The amount of camphor imparting camphoreous, minty and herbal odors [22,23] was lower than 0.30% in the three samples and met the needs of international standards with the amount below 0.5% in lavender flavoring [24]. Only cumaldehyde with spicy odor [25] was identified in aldehydes and the amount was less than 1% in each sample.

#### 2.1.2. Effect of Extraction Solvent on the Odor Characteristics of LEs

**Figure 2:**
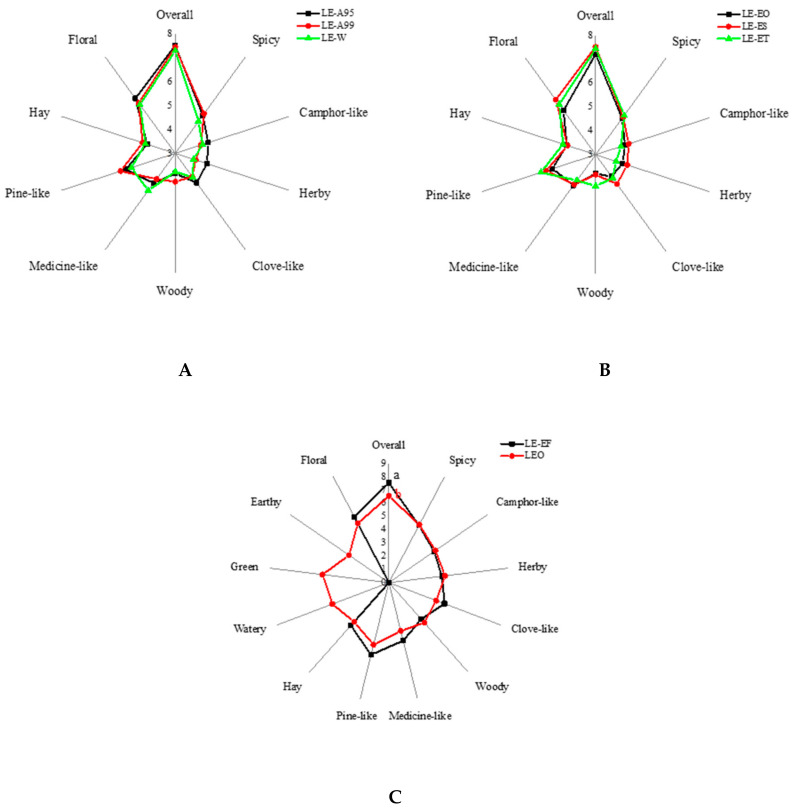
The odor profiles of lavender extracts and lavender essential oils. (**A**) Radar of sensory aroma attribute profile in lavender extracts with different extraction solvents. (**B**) Radar of sensory aroma attribute profile in lavender extracts with different extraction times. (**C**) Radar of sensory aroma attribute profile in lavender essential oil and lavender extracts under the optimized extraction conditions.

In order to describe the actual odor profiles of the lavender extracts, the odor characteristics of LE-W, LE-A95 and LE-A99 were assessed by sensory panelists. As can be seen from Figure 2A, LE-W had the strong intensity of floral, moderate spicy, camphor-like, clove-like, medicine-like, pine-like, herbal and woody odors, while LE-A99 had a stronger intensity of spicy, woody and pine-like odors and a weaker intensity of medicine-like odor. There were no significant differences on the odor attributes of camphor-like, herbal, clove-like and hay odors between LE-W and LE-A99. By comparison, LE-A95 had the highest intensity of floral, camphor-like, herbal and clove-like odors among the three samples, whereas the least intensity of hay odor was found in LE-A95. Other than these findings, no significant difference was found for the other four odor attributes between LE-A95 and LE-A99. Nevertheless, LE-W, in general, tended to have the lowest score of overall assessment. As a strong polar solvent, purified water was weak in dissolving volatile or semi-volatile components. Consistent with the results of volatile compositions, the least odor property might be interpreted as the lowest amount of volatiles in LE-W.

Taken as a whole, the results of sensory evaluation and aroma compositions showed that 95% edible ethanol, which was of lower cost than 99% edible ethanol and had better solubility of volatile and semi-volatile components, could be used as an appropriate extraction solvent to prepare lavender extracts, allowing for the higher intensity of characteristic floral, spicy and herbal odors, better odor property and much more volatile compounds. For the subsequent assays, 95% edible ethanol was selected as the extraction solvent.

#### 2.1.3. Effect of Extraction Times on the Volatile Compositions of LEs

In total, thirty-four volatiles were identified in these samples, and a number of 32, 33 and 32 aroma compounds were found in LE-EO (extraction once), LE-ES (extraction twice) and LE-ET (extraction three times), respectively, and the total amounts of volatiles in each sample were 94.98%, 96.40% and 91.09%, respectively (Figure 1B). Among the identified components were alkenes, alcohols, ketones, esters, aldehydes and a small number of other chemical components.

Similar to the case of LE-A95, as shown in Table 2, the majority of these volatiles were esters; eight aroma compounds were identified, accounting for more than 63.00% of the total amounts of volatiles in each sample, among which linalyl acetate and lavandulyl acetate were the two abundant volatiles. Secondly, the amounts of alcohols and alkenes were abundant, accounting for more than 7.00% and 15.00% of the total amounts of volatiles in each sample, respectively. Similar to LE-A95, linalool and its oxides (I and II) with floral notes were of abundance in each sample; lavandulol imparting floral odor reminiscent of lavender [26] was identified in these samples, and it was the present in the highest amount (1.50%) in LE-EO. Moreover, α-santalene imparting woody and herbal flavors [27] was only found in LE-EO, whereas 1-octen-3-ol was only identified in LE-ES. The amounts of camphor met the requirements of international standards, and the highest and lowest amounts were observed in LE-ET and LE-ES, respectively (0.27% and 0.15%, respectively).

#### 2.1.4. Effect of Extraction Times on the Odor Characteristic of LEs

As shown in Figure 2B, the aroma spectra of LE-EO, LE-ES and LE-ET were significantly different especially for the woody, clove-like, herbal and camphor-like notes. The LE-ES had the higher intensity of clove-like, herbal and camphor-like odors as well as the lower intensity of woody odor. The lowest intensity of hay odor was found in LE-ES. In addition, the intensity of pine-like and spicy odors was enhanced with an increase in extraction times, but no significant difference was observed in the two attributes among the three samples. It is known that the floral note is the characteristic odor attribute for lavender-related products, and the highest intensity of floral odor was found in LE-ES, indicating that twice extraction was beneficial for the preparation of high-quality lavender extracts. What was more, LE-ES had the highest score of overall assessment.

Considering the aroma characteristics, the amounts of volatiles as well as the costs, twice extraction was appropriate for preparing the lavender extracts. Given the fact that linalyl acetate, lavandulyl acetate, lavandulol, linalool and its oxides were of abundance in lavender extracts as well as camphor was staple for the quality of lavender-related products, they were selected for further comparison between the lavender extracts and essential oil.

#### 2.1.5. Verification Experiments on the Preparation of LEs

The verification experiments on the preparation of lavender extracts were conducted twice by adding 95% edible ethanol, for three replicates. Accordingly, the sensory evaluation and aroma analysis of final lavender extracts (LE-EF) were carried out. As can be seen from Figure 2C, LE-EF had the strong intensity of pine-like and floral odors, and moderate spicy, camphor-like, herbal, clove-like, medicine-like, hay and woody notes. No undesired odor was found in LE-EF. In addition, the LE-EF tended to contain a higher overall evaluation score (7.53) than that of other lavender extracts. Furthermore, 32 aroma compounds were identified in LE-EF, including linalyl acetate (46.76%), lavandulyl acetate (14.21%), lavandulol (1.54%), linalool (16.82%), linalool oxide I (0.90%), and linalool oxide II (1.20%), accounting for 97.84% of the total amount of volatiles, and the relative content of camphor was lower than 0.15%, as shown in Table 3. Generally, the results of verification experiments showed that the high-quality lavender extracts could be produced under the optimal extraction conditions, that is, twice extraction with 95% edible ethanol as the extraction solvent.

### 2.2. Odor Property of Lavender Essential Oil

The lavender essential oil (LEO) was obtained by SD, and the mean value of the yields was 2.21% (g/g), which was much higher than that of Dong et al. (2020) [1]. In accordance with lavender extracts, the sensory evaluation and volatile analysis using GC-MS technology were applied to reveal the odor characteristics of lavender essential oil.

In total, forty-eight volatiles were identified, accounting for 96.53% of the total amounts of volatiles in LEO. The majority of these identified volatiles were alcohols (38.14%), esters (37.41%), alkenes (17.28%), ketones (1.18%) and a small number of aromatics (0.48%), aldehydes (0.28%) and heterocyclic compounds (0.02%). As can be seen in Figure 1C, the amounts of alkenes and alcohols in LEO were significantly higher than those in LE-EF, while the relative content of esters was much lower in LEO. Similar to the cases of LE, results obtained from Table 3 showed that linalyl acetate and lavandulyl acetate were the two most abundant ester compounds, accounting for 27.33% and 7.00% of the total amounts of volatiles, respectively, but were much lower than those in LE-EF. Linalool was the most abundant volatile in lavender essential oil with a relative content of 29.01%, which was 1.72 times more than that found in LE-EF. No significant difference was found on the relative content of lavandulol between LEO and LE-EF. In contrast, the relative contents of linalool oxide I and linalool oxide II were lower than 0.50% in LEO, suggesting that they might have little contribution for the floral note of LEO. There were two alkene compounds with a relative content above 3.0%, namely (*E*)-β-ocimene and (*E*)-β-caryophyllene, imparting herbal and woody odors [28], respectively. The relative content of camphor was 0.38% in LEO, which was 2.92 times more than that found in LE-EF. However, the amount of camphor in LEO was far less than that observed in essential oil from Spanish lavender ranging from 10–18% [3], which might be due to the different varieties used. In addition, 1-hexanol with green odor [29], α-thujene with woody note [30], α-pinene and β-pinene imparting woody or herbal odors [31], camphene with woody flavor [32], nerol with citrus and floral odors [33], α-humulene with woody odor [34] and *p*-cymene as well as *o*-cymene were identified in LEO, but not in lavender extracts. Nerol oxide with green, leaf and floral odors [35] was detected in lavender essential oil for the first time.

A total of twenty-five volatiles were commonly identified in LEO and LE-EF, including limonene, (*E*)-β-ocimene, (*Z*)-β-ocimene, linalool and its oxides (I and II), camphor, lavandulol, (*E*)-β-caryophyllene and (*E*)-β-farnesene, among which linalool and lavandulyl acetate imparting floral notes were the most abundant volatiles with relative contents higher than 10.0%. In addition, the essential oils contained significantly lower amounts of linalyl acetate, lavandulyl acetate and linalool oxides (I and II) than those of the lavender extracts, whereas the amounts of linalool and camphor were significantly higher than that in LE-EF.

The LEO had a strong floral note, and moderate spicy, camphor-like, herbal, woody, pine-like, clove-like, hay and medicine-like odors (Figure 2C). The intensity of camphor-like, herbal and woody odors were higher than those in LE-EF, while the remaining attributes were opposite. In addition, the aroma attributes of watery and green notes with strong intensity as well as moderate earthy odor were only found in LEO. 1-Hexanol might be the contributor for the green note in LEO, and the significantly stronger intensity of camphor-like odor might be from the contribution of camphor with a higher amount in LEO. Nevertheless, LEO had a significantly lower score of overall assessment than that of LE-EF, which might be due to the influence of the undesired odor of the watery note.

### 2.3. Principal Component Analysis

**Figure 3:**
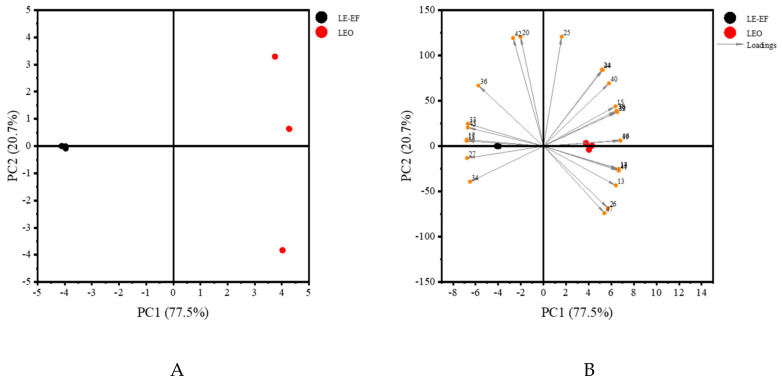
Principal component analysis (PCA) of lavender extracts and lavender essential oil. (**A**) The score plot of PCA analysis. (**B**) The biplot of PCA analysis. Compound numbers correspond to Table 3.

To further distinguish the differences between lavender essential oil and lavender extracts, principal component analysis (PCA) was conducted on the common volatiles, which were the same volatiles identified in LEO and LE-EF. According to the PCA analysis in Figure 3A, the accumulated contribution rate of the first two principal components (PC 1, 77.5%; PC 2, 20.7%) was 98.2%, which appeared to represent the sufficient information of samples, and the two samples were well-separated. The LE-EF exhibited high scores on negative PC 1, where the loadings of characteristic volatile compounds including 1-octen-3-yl acetate, *trans*-α-bergamotene, lavandulyl acetate, carvone, coumarin, linalool oxide I, linalool oxide II, cryptone and linalyl acetate were high, among which linalyl acetate was the most characteristic aroma compound in lavender extracts, and most of them imparted a floral note. The LEO showed high scores in positive PC 1, which contained high loadings of compounds, including lavandulol, camphol, (*E*)-β-farnesene, α-santalene, caryophyllene oxide, terpinen-4-ol, 1,8-cineole, limonene—the characteristic aroma compounds with camphor-like, spicy or herbal odors in lavender essential oils (Figure 3B).

## 3. Materials and Methods

### 3.1. Reagents and Materials

The lavender samples were purchased from Yili Derun Agricultural Development Co., Ltd., Yili City, China. The inflorescences of *L. angustifolia* Mill. were harvested during the full blooming period in June 2018, then dried and stored at room temperature. Deionized water was prepared by a Milli-Q water purification system (Millipore, Billerica, MA, USA). All authentic standards were obtained from Sigma Aldrich (Shanghai, China), unless specified otherwise. 

*n*-Alkanes (C6–C25) were purchased from Supelco, Bellefonte, PA, USA. Edible ethanol (95% and 99%) was purchased from Taicang Xintai Alcohol Co., Ltd., Suzhou, China. Sodium sulfate (Sinopharm Chemical Reagent Co., Ltd., Shanghai, China) and all other chemicals were of analytical grade. 

### 3.2. Essential Oil Isolation

The steam distillation (SD) method was from Cai et al. (2013) [14] with slight modification, and Clevenger apparatus was used for essential oil preparation. Dried lavender flowers (100 g) were placed into a 2000 mL flask with 1500 mL of purified water. The distillation lasted for 1.5 h until thick liquid was extracted no more, the upper layer of essential oil was transferred to a separatory funnel after cooling at room temperature and then dried overnight using anhydrous sodium sulfate. The essential oil was maintained at 4 °C for the subsequent assays. The isolation of essential oil was replicated three times.

### 3.3. Lavender Extracts Preparation with Different Extraction Solvents

The preparation of lavender extracts was performed according to Guo et al. (2018) [13] with minor modifications. Briefly, the extraction was conducted twice by adding 300 g of different extraction solvents to 50 g of lavender flowers in a 1000 mL beaker which was sealed with foil. The mixture was static-extracted at 60 °C for 2 h using a water bath (DK-S22, Shanghai Jing Hong Laboratory Instrument Co., Ltd., Shanghai, China). The residues were removed by filtration. After cooling, the filtrates were combined and concentrated to about 50 g by using a rotary vacuum evaporator (R-210, Buchi Labortechnik AG, Flawil, Switzerland). After centrifugation at 5000 rpm for 15 min (SL8, Thermo Fisher Scientific Inc., Waltham, MA, USA), the supernatant was obtained and diluted with an extraction solvent to 50 g. Three kinds of extraction solvents were used, which were purified water and 95% and 99% edible ethanol; accordingly, the names of the lavender extracts were LE-W, LE-A95 and LE-A99, respectively. All experiments were replicated three times.

### 3.4. Lavender Extract Preparation with Different Extraction Times

The effects of extraction times on odor characteristics of lavender extracts were studied with 95% edible ethanol as the extraction solvent. Similar to the method described in Section 3.3, the lavender extracts were prepared by adding 95% edible ethanol with different extraction treatment times, which were once, twice and three times; accordingly, the names of lavender extracts were LE-EO, LE-ES and LE-ET, respectively. The extracts were kept in 4 °C for the subsequent assays. All experiments were replicated three times.

### 3.5. Sensory Evaluation

A sensory analysis was performed according to Guo et al. (2021) [36] with modifications. The lavender extracts and essential oil were prepared according to the methods mentioned above, and then each sample was subjected to a sensory test. Three-digit numbers were used to code samples and they were randomly offered to panelists. The intensity values and aroma descriptors of samples were recorded by panelists. Panelists agreed that the odor of the lavender samples could be described using attributes such as floral, hay, pine-like, medicine-like, woody, clove-like, herbal, camphor-like and spicy. Besides that, the attributes of earthy, green and watery notes were special for lavender essential oil. The intensities of the aroma attributes were scored using a scale from 0 to 10, the higher the score, the stronger the intensity, where 0 = none or not perceptible intensity, 3 = weak intensity, 5 = moderate intensity, 7 = high intensity and 10 = extremely high intensity. Each sample was evaluated three times by each panelist on different days. Data were expressed as mean values.

The lavender samples were evaluated by a well-trained panel of thirteen members (seven males and six females, age from 24–40 years). All assessors had more than two years of experience in the descriptive sensory analysis of natural plant extracts and essential oils. Panelists were trained using a series of important aroma compounds.

### 3.6. Determination of Volatile Compounds in Lavender Extracts

The SPME method was modified from Guo et al. (2019) [15] and applied for the extraction of volatile compounds in lavender extracts. The lavender extracts (0.05 g) were placed into a 20 mL headspace vial which was sealed with a cap. An SPME fiber (50/30 μm DVB/CAR/PDMS (Divinylbenzene/Carboxen/Polydimethylsiloxane), Supelco Inc., Bellefonte, PA, USA) was exposed to the sample headspace at 50 °C to collect volatile compounds for 30 min. The volatiles were desorbed by inserting the SPME fiber into a GC injector with a split ratio of 10:1, connected with a fused-silica GC column (DB-5, 30 m × 0.25 mm × 0.25 μm, J&W Scientific, Folsom, CA, USA) for 5 min. The volatile compositions of lavender extracts were analyzed by GC-MS (Agilent 7890A-5975C inert mass spectrometer detector (MSD), Agilent, Santa Clara, CA, USA). The initial temperature of the GC was set at 50 °C for 5 min; increased at a rate of 2 °C min^−1^ to 160 °C, maintained for 5 min; and then raised to 250 °C at 12 °C min^−1^, held for 5 min. The detector temperature was set at 250 °C. Helium gas (37 cm/s) was used as a mobile phase. The mass spectrometer was operated at electron ionization mode (positive ion, 70 eV). Ion source and transfer-line temperatures were 200 and 150 °C, respectively. The mass spectra were acquired in the full-scan mode from 30–500 amu. Retention indices (RIs) were calculated from the retention time of *n*-alkanes by linear interpolation.

### 3.7. Determination of Volatile Compounds in Lavender Essential Oil

The constituents of lavender essential oil were analyzed by GC-MS (Agilent 7890A-5977A inert MSD, Agilent, Santa Clara, CA, USA). Separation was achieved by a fused-silica capillary column (HP-5 MS, 30 m × 0.25 mm × 0.25 μm, J&W, Folsom, CA, USA). The temperature program was 65 °C, held for 1 min; increased to 90 °C at a rate of 5 °C min^−1^ and retained 1 min; and raised to 260 °C at 10 °C min^−1^. Injection volume was 1.0 μL with a split ratio of 100:1 for lavender essential oil. The detector temperature was set at 270 °C and helium gas (37 cm/s) was used as a mobile phase. The setting conditions of mass spectrometer were the same as that described in Section 3.6.

### 3.8. The Analysis of Volatile Compositions

A comprehensive analysis of aroma compositions in lavender samples by GC-MS, using the computer NIST 2017 (National Institute of Standards and Technology, USA; 17th edition) database, combining with the retention time, retention index, in-house database and literature data [37,38] and also using advanced peak deconvolution and data processing software, revealed many overlapping compounds, if available, positively identified with those of authentic compounds. The amounts of the identified compounds were calculated as the percentage of the total peak areas (%).

### 3.9. Statistical Analysis

All experiments were replicated three times. The statistical analysis was performed with one-way analysis of variance (ANOVA) by using SPSS Statistics software, version 22 (IBM, Armonk, New York, NY, USA). All comparisons were considered statistically significant if *p* value < 0.05.

## 4. Conclusions

The present study demonstrated that extraction solvent and extraction times had an important effect on the flavor quality of lavender extracts, which could be judged by the volatile compositions, total volatiles and sensory evaluation. The lavender extracts were prepared and the optimum conditions were found to be extraction two times with 95% edible ethanol as the extraction solvents, allowing for lavender extracts with high amounts of volatiles, total volatiles and strong intensity of floral, clove-like and herbal odors as well as high scores of overall assessment. In addition, the contrastive analysis on aroma properties of lavender extracts and essential oil was performed. The esters, alcohols and alkenes compounds were the main volatiles in both lavender extracts and essentials oil, but the number and proportion of the volatile categories were significantly different between them. LEO and LE-EF tended to contain a higher proportion of esters and alcohols, while the largest numbers of volatiles were found in alkenes. The higher intensity of aroma attributes, which were herbal, woody and camphor-like odors, was found in LEO, whereas the LE-EF had a significantly higher intensity of floral note than that in LEO. The earthy, green and watery odors were only recorded in LEO. Furthermore, LE-EF had a much higher score of overall assessment than that of LEO. Though much more volatile compounds were detected in LEO, the relative content of most volatiles was lower than that in LE-EF, such as linalool oxide I and II, linalyl acetate and lavandulyl acetate, whereas the LEO contained higher amounts of linalool and camphor, which might be the contributor for the higher intensity of earthy odor in LEO. Nerol oxide imparting green or floral odors was identified in lavender-related products for the first time. On the whole, the lavender extracts had better odor properties with strong herbal, clove-like and floral odors reminiscent of lavender in comparison to lavender essential oil. However, the comparative applications of lavender extracts and essential oil in food or beverage need further study. In summary, the scientific results offered the theoretical basis for the preparation of lavender extracts and their industrial production, and also provided reference for the study on aroma properties and application performance of lavender-related products.

## Figures and Tables

**Table 1:** Volatile compositions of lavender extracts with different extraction solvents.

No.	RT	RI	Volatile Compounds	Relative Content (%)			ID *
				LE-A95	LE-A99	LE-W	
1	2.44	666	Acetic acid	0.22 ± 0.01	0.71 ± 0.00	0.59 ± 0.02	MS, RI, S
2	9.35	979	1-Octen-3-ol	0.05 ± 0.00	nd	nd	MS, RI, S
3	9.66	988	β-Myrcene	0.89 ± 0.00	0.63 ± 0.02	0.56 ± 0.02	MS, RI, S
4	11.39	996	Hexyl acetate	0.53 ± 0.03	0.18 ± 0.00	0.12 ± 0.00	MS, RI, S
5	12.19	1018	Limonene	0.15 ± 0.01	0.07 ± 0.00	0.06 ± 0.00	MS, RI, S
6	13.37	1022	1,8-Cineole	2.25 ± 0.10	1.26 ± 0.00	0.58 ± 0.04	MS, RI
7	19.52	1048	(*E*)-β-Ocimene	0.86 ± 0.00	0.25 ± 0.01	0.19 ± 0.01	MS, RI, S
8	20.04	1052	(*Z*)-β-Ocimene	0.71 ± 0.02	0.38 ± 0.01	0.32 ± 0.02	MS, RI, S
9	21.46	1081	Linalool oxide Ⅱ ((*E*)-furanoid)	1.13 ± 0.01	1.08 ± 0.05	0.51 ± 0.01	MS, RI, S
10	22.72	1098	Linalool oxide Ⅰ ((*Z*)-furanoid)	0.92 ± 0.04	0.90 ± 0.04	0.39 ± 0.01	MS, RI, S
11	23.75	1111	1-Octen-3-yl acetate	3.93 ± 0.16	2.72 ± 0.04	1.95 ± 0.03	MS, RI
12	23.83	1120	Camphol	0.28 ± 0.02	0.44 ± 0.02	0.48 ± 0.02	MS, RI, S
13	24.40	1145	Camphor	0.15 ± 0.00	0.27 ± 0.00	0.20 ± 0.00	MS, RI
14	24.96	1159	Cryptone	0.48 ± 0.00	0.71 ± 0.02	0.71 ± 0.05	MS, RI
15	26.03	1159	Terpinen-4-ol	0.16 ± 0.00	0.24 ± 0.01	0.28 ± 0.01	MS, RI
16	29.68	1163	Linalool	12.51 ± 0.72	11.63 ± 0.29	7.72 ± 0.41	MS, RI, S
17	30.16	1165	(3*E*,5*Z*)-1,3,5-Undecatriene	0.33 ± 0.02	0.13 ± 0.01	0.10 ± 0.00	MS, RI
18	30.18	1166	Linalyl acetate	46.62 ± 0.45	47.22 ± 1.01	47.00 ± 1.14	MS, RI
19	30.59	1170	Lavandulol	0.61 ± 0.03	0.91 ± 0.03	1.11 ± 0.05	MS, RI
20	30.89	1192	Hexyl butanoate	0.68 ± 0.03	0.51 ± 0.01	0.42 ± 0.01	MS, RI
21	31.10	1238	Carvone	0.07 ± 0.00	0.09 ± 0.00	0.14 ± 0.00	MS, RI
22	31.92	1247	Cumaldehyde	0.35 ± 0.01	0.39 ± 0.00	0.46 ± 0.02	MS, RI
23	32.91	1268	Lavandulyl acetate	11.88 ± 0.14	11.10 ± 0.36	10.32 ± 0.11	MS, RI
24	34.86	1289	Bornyl acetate	0.34 ± 0.00	0.47 ± 0.03	0.40 ± 0.02	MS, RI
25	35.90	1348	Copaene	0.16 ± 0.01	0.15 ± 0.01	0.10 ± 0.01	MS, RI, S
26	36.63	1363	Neryl acetate	0.30 ± 0.02	0.26 ± 0.01	0.26 ± 0.01	MS, RI, S
27	37.02	1383	Geranyl acetate	0.57 ± 0.03	0.45 ± 0.01	0.44 ± 0.01	MS, RI, S
28	37.32	1433	*trans-*α-Bergamotene	0.54 ± 0.02	0.47 ± 0.00	0.54 ± 0.01	MS, RI
29	38.60	1442	(*E*)-β-Caryophyllene	6.67 ± 0.14	4.7 ± 0.14	5.02 ± 0.07	MS, RI, S
30	39.18	1469	(*E*)-β-Farnesene	1.37 ± 0.06	1.37 ± 0.08	1.85 ± 0.11	MS, RI, S
31	40.85	1477	Coumarin	0.13 ± 0.01	0.14 ± 0.00	0.24 ± 0.01	MS, RI, S
32	41.18	1484	Germacrene D	0.31 ± 0.02	0.30 ± 0.01	0.13 ± 0.00	MS, RI
33	50.78	1593	Caryophyllene oxide	0.25 ± 0.00	0.29 ± 0.01	0.41 ± 0.02	MS, RI

* Identification method; MS, identification based on the NIST 2017 (National Institute of Standards and Technology, USA; 17th edition) mass spectral database; RT, retention time; RI, retention index; S, the compounds were identified using authentic standard compounds; nd, not detectable. LE-W—extraction with purified water; LE-A95—extraction with 95% edible ethanol; LE-A99—extraction with 99% edible ethanol.

**Table 2:** Volatile compositions of lavender extracts with different extraction times and verification experiments.

No.	RT	RI	Volatile Compounds	Relative Content (%)				ID *
				LE-EO	LE-ES	LE-ET	LE-EF	
1	2.44	666	Acetic acid	0.48 ± 0.03	0.22 ± 0.01	0.67 ± 0.03	0.28 ± 0.01	MS, RI, S
2	9.35	979	1-Octen-3-ol	nd	0.05 ± 0.00	nd	0.15 ± 0	MS, RI, S
3	9.66	988	β-Myrcene	0.60 ± 0.00	0.89 ± 0.00	0.65 ± 0.02	0.83 ± 0.02	MS, RI, S
4	11.39	996	Hexyl acetate	0.12 ± 0.01	0.53 ± 0.03	0.19 ± 0.01	0.01 ± 0	MS, RI, S
5	12.19	1018	Limonene	0.06 ± 0.00	0.15 ± 0.01	0.07 ± 0.01	0.09 ± 0	MS, RI, S
6	13.37	1022	1,8-Cineole	0.45 ± 0.02	2.25 ± 0.10	1.27 ± 0.02	0.18 ± 0	MS, RI
7	19.52	1048	(*E*)-β-Ocimene	0.20 ± 0.01	0.86 ± 0.00	0.25 ± 0.00	0.28 ± 0.01	MS, RI, S
8	20.04	1052	(*Z*)-β-Ocimene	0.35 ± 0.00	0.71 ± 0.02	0.41 ± 0.03	0.48 ± 0.01	MS, RI, S
9	21.46	1081	Linalool oxide Ⅱ ((*E*)-furanoid)	1.24 ± 0.04	1.13 ± 0.01	1.23 ± 0.09	1.2 ± 0.06	MS, RI, S
10	22.72	1098	Linalool oxide Ⅰ ((*Z*)-furanoid)	1.00 ± 0.09	0.92 ± 0.04	0.91 ± 0.01	0.9 ± 0.03	MS, RI, S
11	23.75	1111	1-Octen-3-yl acetate	2.00 ± 0.06	3.93 ± 0.16	2.68 ± 0.04	1.31 ± 0	MS, RI
12	23.83	1120	Camphol	0.71 ± 0.02	0.28 ± 0.02	0.43 ± 0.01	0.74 ± 0	MS, RI, S
13	24.40	1145	Camphor	0.24 ± 0.02	0.15 ± 0.00	0.27 ± 0.00	0.13 ± 0	MS, RI
14	24.96	1159	Cryptone	0.80 ± 0.03	0.48 ± 0.00	0.73 ± 0.03	0.81 ± 0.03	MS, RI
15	26.03	1159	Terpinen-4-ol	0.35 ± 0.02	0.16 ± 0.00	0.22 ± 0.02	0.39 ± 0	MS, RI
16	29.68	1163	Linalool	14.58 ± 0.18	7.72 ± 0.41	11.87 ± 0.34	16.82 ± 0.31	MS, RI, S
17	30.16	1165	(3*E*,5*Z*)-1,3,5-Undecatriene	nd	0.33 ± 0.02	0.13 ± 0.00	nd	MS, RI
18	30.18	1166	Linalyl acetate	47.28 ± 0.35	46.62 ± 0.45	47.08 ± 0.19	46.76 ± 0.37	MS, RI
19	30.59	1170	Lavandulol	1.50 ± 0.06	0.61 ± 0.03	0.86 ± 0.04	1.54 ± 0.01	MS, RI
20	30.89	1192	Hexyl butanoate	0.48 ± 0.02	0.68 ± 0.03	0.52 ± 0.01	0.37 ± 0.01	MS, RI
21	31.10	1238	Carvone	0.17 ± 0.02	0.07 ± 0.00	0.09 ± 0.01	0.19 ± 0	MS, RI
22	31.92	1247	Cumaldehyde	0.63 ± 0.00	0.35 ± 0.01	0.38 ± 0.01	0.63 ± 0.01	MS, RI
23	32.91	1268	Lavandulyl acetate	13.46 ± 0.09	11.88 ± 0.14	12.05 ± 0.82	14.21 ± 0.28	MS, RI
24	34.86	1289	Bornyl acetate	0.46 ± 0.03	0.34 ± 0.00	0.45 ± 0.03	0.5 ± 0.01	MS, RI
25	35.90	1348	Copaene	0.07 ± 0.00	0.16 ± 0.01	0.15 ± 0.00	nd	MS, RI, S
26	36.63	1363	Neryl acetate	0.33 ± 0.01	0.30 ± 0.02	0.25 ± 0.01	0.41 ± 0.02	MS, RI, S
27	37.02	1383	Geranyl acetate	0.49 ± 0.04	0.57 ± 0.03	0.44 ± 0.01	0.68 ± 0.01	MS, RI, S
28	37.11	1402	α-Santalene	0.96 ± 0.08	nd	nd	0.5 ± 0.01	MS, RI
29	37.32	1433	*trans*-α-Bergamotene	0.32 ± 0.01	0.54 ± 0.02	0.50 ± 0.04	0.24 ± 0.01	MS, RI
30	38.60	1442	(*E*)-β-Caryophyllene	3.34 ± 0.15	6.67 ± 0.14	4.33 ± 0.37	2.34 ± 0.01	MS, RI, S
31	39.18	1469	(*E*)-β-Farnesene	1.24 ± 0.02	1.37 ± 0.06	1.31 ± 0.05	1.13 ± 0.01	MS, RI, S
32	40.85	1477	Coumarin	0.27 ± 0.02	0.13 ± 0.01	0.14 ± 0.00	0.17 ± 0.01	MS, RI, S
33	41.18	1484	Germacrene D	0.30 ± 0.01	0.31 ± 0.02	0.29 ± 0.02	0.09 ± 0	MS, RI
34	50.78	1593	Caryophyllene oxide	0.50 ± 0.04	0.25 ± 0.00	0.28 ± 0.02	0.63 ± 0	MS, RI

* Identification method; MS, identification based on the NIST 2017 mass spectral database; RT, retention time; RI, retention index; S, the compounds were identified using authentic standard compounds; nd, not detectable.

**Table 3:** Volatile compositions of lavender essential oils.

No.	RT	RI	Volatile Compounds	LEO (%)	ID *
1	2.56	748	3-Methyl-2-butenal	0.01 ± 0.00	MS, RI, S
2	3.49	852	1-Hexanol	0.05 ± 0.00	MS, RI, S
3	4.54	929	α-Thujene	0.10 ± 0.06	MS, RI
4	4.65	931	α-Pinene	0.28 ± 0.04	MS, RI, S
5	4.95	958	Camphene	0.33 ± 0.09	MS, RI, S
6	5.46	963	1-Octen-3-ol	0.47 ± 0.08	MS, RI, S
7	5.65	989	3-Octanone	0.28 ± 0.18	MS, RI
8	5.74	990	β-Pinene	0.70 ± 0.05	MS, RI, S
9	6.23	1003	3-Carene	0.78 ± 0.06	MS, RI
10	6.47	1011	*p*-Cymene	0.13 ± 0.03	MS, RI, S
11	6.54	1015	*o*-Cymene	0.35 ± 0.01	MS, RI, S
12	6.65	1018	Limonene	0.95 ± 0.18	MS, RI, S
13	6.73	1022	1,8-Cineole	1.02 ± 0.27	MS, RI
14	6.85	1034	(*E*)-β-Ocimene	4.38 ± 0.91	MS, RI, S
15	7.13	1036	(*Z*)-β-Ocimene	1.71 ± 0.37	MS, RI, S
16	7.44	1047	γ-Terpinene	0.09 ± 0.04	MS, RI, S
17	8.21	1081	Linalool oxide Ⅱ ((*E*)-furanoid)	0.22 ± 0.07	MS, RI, S
18	7.83	1098	Linalool oxide Ⅰ ((*Z*)-furanoid)	0.28 ± 0.03	MS, RI, S
19	8.68	1103	Linalool	29.01 ± 0.33	MS, RI, S
20	8.79	1106	1-Octen-3-yl acetate	1.25 ± 0.18	MS, RI
21	9.15	1118	*allo*-Ocimene	0.33 ± 0.09	MS, RI
22	9.53	1121	Camphor	0.38 ± 0.03	MS, RI
23	9.68	1137	Nerol oxide	0.02 ± 0.01	MS, RI
24	9.98	1161	Camphol	1.65 ± 0.66	MS, RI
25	10.00	1162	Lavandulol	1.72 ± 0.71	MS, RI, S
26	10.20	1167	Terpinen-4-ol	2.24 ± 1.05	MS, RI
27	10.39	1170	Cryptone	0.31 ± 0.04	MS, RI
28	10.54	1172	α-Terpineol	2.03 ± 0.22	MS, RI, S
29	10.81	1196	Eucarvone	0.08 ± 0.05	MS, RI
30	10.99	1226	(*Z*)-Carveol	0.03 ± 0.00	MS, RI
31	11.15	1237	Nerol	0.40 ± 0.08	MS, RI
32	11.37	1239	α-Methylbenzenepropanal	0.23 ± 0.03	MS, RI
33	11.44	1240	Carvone	0.04 ± 0.03	MS, RI
34	11.68	1251	Linalyl acetate	27.33 ± 5.05	MS, RI, S
35	12.02	1280	Phellandral	0.04 ± 0.01	MS, RI
36	12.25	1294	Lavandulyl acetate	7.00 ± 3.86	MS, RI
37	12.31	1295	Cuminol	0.04 ± 0.01	MS, RI
38	13.33	1367	Neryl acetate	0.60 ± 0.06	MS, RI
39	13.63	1383	Geranyl acetate	1.23 ± 0.15	MS, RI, S
40	14.21	1418	α-Santalene	0.77 ± 0.15	MS, RI
41	14.26	1421	(*E*)-β-Caryophyllene	3.67 ± 0.26	MS, RI, S
42	14.41	1427	*trans-*α-Bergamotene	0.22 ± 0.05	MS, RI
43	14.46	1428	Coumarin	0.08 ± 0.02	MS, RI, S
44	14.64	1455	(*E*)-β-Farnesene	1.66 ± 0.38	MS, RI, S
45	14.73	1456	α-Humulene	0.20 ± 0.00	MS, RI
46	15.09	1482	Germacrene D	0.80 ± 0.02	MS, RI
47	16.44	1561	Caryophyllene oxide	0.72 ± 0.06	MS, RI
48	17.06	1589	epi-Bicyclosesquiphellandrene	0.29 ± 0.12	MS, RI

* Identification method; MS, identification based on the NIST 2017 mass spectral database; RT, retention time; RI, retention index; S, the compounds were identified using authentic standard compounds.
